# Classification of Benign and Malignant Breast Tumors Using H-Scan Ultrasound Imaging

**DOI:** 10.3390/diagnostics9040182

**Published:** 2019-11-08

**Authors:** Yali Ouyang, Po-Hsiang Tsui, Shuicai Wu, Weiwei Wu, Zhuhuang Zhou

**Affiliations:** 1 Department of Biomedical Engineering, College of Life Science and Bioengineering, Beijing University of Technology, Beijing 100124, China; ouyangg@emails.bjut.edu.cn (Y.O.); wushuicai@bjut.edu.cn (S.W.); 2 Department of Medical Imaging and Radiological Sciences, College of Medicine, Chang Gung University, Taoyuan 33302, Taiwan; tsuiph@mail.cgu.edu.tw; 3 Department of Medical Imaging and Intervention, Chang Gung Memorial Hospital at Linkou, Taoyuan 33302, Taiwan; 4 Medical Imaging Research Center, Institute for Radiological Research, Chang Gung University and Chang Gung Memorial Hospital at Linkou, Taoyuan 33302, Taiwan; 5 College of Biomedical Engineering, Capital Medical University, Beijing 100069, China; wuweiwei8889@163.com

**Keywords:** breast cancer, H-scan ultrasound imaging, Hermite polynomial, scatterer size, ultrasound tissue characterization

## Abstract

Breast cancer is one of the most common cancers among women worldwide. Ultrasound imaging has been widely used in the detection and diagnosis of breast tumors. However, due to factors such as limited spatial resolution and speckle noise, classification of benign and malignant breast tumors using conventional B-mode ultrasound still remains a challenging task. H-scan is a new ultrasound technique that images the relative size of acoustic scatterers. However, the feasibility of H-scan ultrasound imaging in the classification of benign and malignant breast tumors has not been investigated. In this paper, we proposed a new method based on H-scan ultrasound imaging to classify benign and malignant breast tumors. Backscattered ultrasound radiofrequency signals of 100 breast tumors were used (48 benign and 52 malignant cases). H-scan ultrasound images were constructed with the radiofrequency signals by matched filtering using Gaussian-weighted Hermite polynomials. Experimental results showed that benign breast tumors had more red components, while malignant breast tumors had more blue components in H-scan ultrasound images. There were significant differences between the **RGB** channels of H-scan ultrasound images of benign and malignant breast tumors. We conclude H-scan ultrasound imaging can be used as a new method for classifying benign and malignant breast tumors.

## 1. Introduction

Breast cancer is one of the most common malignant tumors in the world and the leading cause of death among women [1,2]. Early detection and diagnosis of breast cancer is of critical importance. At present, biopsy serves as the gold standard for diagnosing breast tumors [3]. Unfortunately, biopsy is an invasive histological examination method with poor repeatability, and has the risk of infection and bruising [4]. Less than 30% of the breast tumors are malignant after surgical biopsy [5]. Breast biopsy is usually performed after getting suspicious results from imaging examination. The primary imaging modalities for detecting breast cancer are mammography and ultrasonography [6]. However, mammography is usually not suitable in 20–50% of patients with dense breasts [7]. Women with glandular breasts are four times more likely to develop breast cancer than women with fatty breast tissue [8]. Ultrasound is a low-cost, real-time, non-ionizing medical imaging modality. Breast density does not inhibit ultrasound waves in the breast, and ultrasound is a safe and useful technique in women [9,10]. However, the current diagnosis based on breast ultrasound is more dependent on observer subjectivity than mammography and magnetic resonance imaging (MRI) [5]. In some cases, it is easy to cause misdiagnosis and missed diagnosis, leading to unnecessary biopsy [11].

The backscattered radiofrequency (RF) signal is modeled as the interaction of an ultrasound pulse with the scattering structure. The properties of scatterers are related to tissue microstructures (less than wavelength) and can be used to characterize the structural changes that are not obvious in the B-mode ultrasound images [12,13]. Several ultrasound tissue characterization techniques have been introduced in previous studies [14,15,16,17,18]. The main limitation of these approaches is that they need calibration steps before measurement, or use relatively large ultrasound data kernels to improve the accuracy of tissue characterization [19]. To overcome the limitation of conventional tissue characterization methods, a new classification method for acoustic scatterers called H-scan has recently emerged [19,20,21,22].

H-scan is a new ultrasound technique that images the relative size of acoustic scatterers. Compared with the conventional tissue characterization techniques, it does not need any statistical averaging to estimate an expected value for some scattering parameters, thus maintaining the axial resolution. This analysis is performed using a class of Gaussian-weighted Hermite polynomials, which have unique properties closely related to the mathematics of ultrasound scattering. By matching the pulse reflection convolution model with the Gaussian-weighted Hermite polynomial, the echo signal can be divided into information returned from a specific class of scatterers. H-scan can effectively display some information hidden in the conventional B-mode ultrasound image. Gary et al. [23] used H-scan ultrasound imaging to analyze the nodules in different thyroid glands; the feasibility of H-scan to distinguish different size scatterers in tissues was demonstrated. Khairalseed et al. [24] showed that H-scan ultrasound imaging is an effective tissue characterization technique and may have prognostic value in monitoring the response of early cancer to anticancer therapy. Tai et al. [25] proposed an adaptive attenuation correction method for H-scan ultrasound imaging using K-means clustering.

However, the feasibility of H-scan ultrasound imaging in the classification of benign and malignant breast tumors has not been investigated. The structural differences between benign and malignant breast lesions may be due to the presence of scatterers with different sizes. The aim of this study is to study the feasibility of H-scan imaging to differentiate between benign and malignant breast tumors. Ultrasound RF data of 100 breast tumors (52 malignant and 48 benign tumors) were used to construct the H-scan images, which were analyzed for tumor classification.

## 2. Theory

During ultrasound imaging, an RF echo signal is modeled as the interaction of an ultrasound pulse with the scattering structure. The RF signal *e*(*t*) is approximated by [20,26]

(1) $$e(t)=A\left\{p(t)s(x,y)***R(x,y,\frac{ct}{2})\right\},$$

where ∗∗∗ represents the three-dimensional (3-D) convolution operator; *A* is an amplitude constant; *p*(*t*) is a propagation pulse in the axial direction *z*; *s*(*x*, *y*) is the beam pattern; *R*(*x*, *y*, *z*) is the 3-D pattern of reflectors or scatterers; *c* is the speed of sound. According to the round trip of echo signal, the axial distance *z* is equal to *ct*/2.

The *n*th order Hermitian polynomials are derived from the successive differential of Gaussian pulse $e^{-t^{2}}$ [20,27]:

(2) $$H_{n}\left(t\right)={\left(-1\right)}^{n}e^{t^{2}}\frac{d^{n}}{dt^{n}}e^{-t^{2}},\;\;\;\;\;\;n=0,1,2\cdot \cdot \cdot ;\;t\in \pm \infty .$$

The Gaussian-weighted Hermite polynomials are obtained by multiplying Equation (2) by an envelope $G=e^{-t^{2}}$. In fact, the **G***H*_4_(*t*) function is similar to the typical broadband ultrasound pulse [20]. Gaussian-weighted Hermite polynomials can be used as a band-pass filter to select different frequency information [19].

Assume that a **G***H*_4_(*t*) pulse is transmitted into a tissue; the pulse is backscattered, received, and convolved with a **G***H_n_* matched filter [23]:

(3) $$m_{n}\left(t\right)=e\left(t\right)*GH_{n}\left(t/\tau \right),$$

where *m_n_*(*t*) is the output of the matched filter; *τ* is a constant scaling factor; **G***H_n_*(*t*/*τ*) is the H-scan channel matched filter assigned to a color.

## 3. Materials and Methods

### 3.1. Dataset

In this study, we used the publicly available breast lesion ultrasound dataset, the open access series of breast ultrasonic data (OASBUD) [28]. The dataset contained raw ultrasound data (before B-mode image reconstruction) recorded from breast focal lesions, among which 52 were malignant and 48 were benign. All malignant lesions were histologically assessed by core needle biopsy. For benign lesions, part of them (*n* = 37) were histologically assessed and another part (*n* = 13) was observed over a 2-year period [28]. All data were acquired with an Ultrasonix SonixTouch Research ultrasound scanner using an L14–5/38 linear array transducer. The center frequency of the imaging pulse was 10 MHz. Single focus beamforming technique was used, and the focal zone was always located at the depth of the lesion. Each RF data matrix consisted of up to 500 RF scan lines. The sampling frequency was 40 MHz, and the number of samples in every RF signal depended on the chosen depth of the examination. For each lesion, the specific individual region of interest (ROI) was determined by an experienced radiologist.

### 3.2. Radiofrequency Signal Processing and H-Scan Ultrasound Imaging

**Figure 1:**
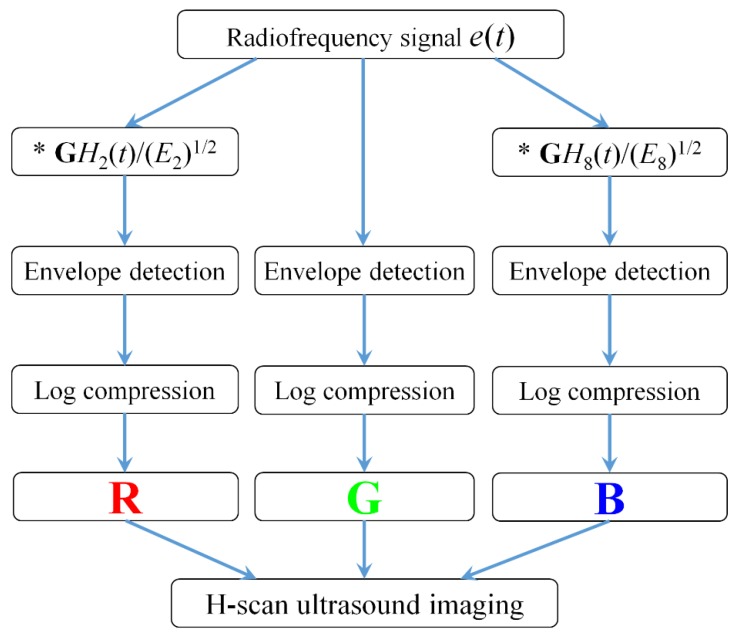
Flow chart of H-scan ultrasound imaging.

Figure 1 shows the algorithmic steps for RF signal processing and H-scan ultrasound imaging. All the data processing was performed in MATLAB 2016b. The RF data *e*(*t*) was used from the OASBUD database. The **G***H*_2_(*t*) and **G***H*_8_(*t*) filtering kernels were set to capture the low and high frequency components of each original RF signal [19,20].

**Figure 2:**
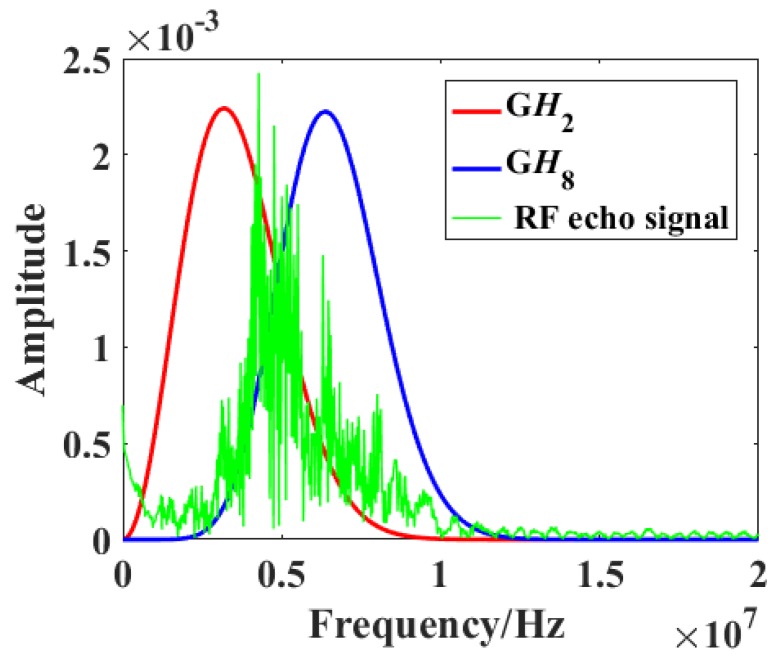
Frequency spectra of the raw radiofrequency (RF) signal (green) and the **G***H*_2_ (red) and **G***H*_8_ (blue) filters.

Figure 2 shows the frequency spectra of a raw RF signal (green) and the **G***H*_2_ (red) and **G***H*_8_ (blue) filters. Two parallel convolution filters were applied to the obtained RF data sequences to measure the relative strength of received signals relative to **G***H*_2_ and **G***H*_8_ after normalization by the signal energy $\sqrt{E_{n}}$, which is defined by [20]

(4) $$E_{n}=\int_{-\infty }^{+\infty }G^{2}H_{n}^{2}dt=1\times 3\times 5\times \cdot \cdot \cdot \times \left|2n-1\right|\times \sqrt{\pi /2}.$$

The envelope for each of these filtered and the original unfiltered signals was calculated using the Hilbert transformation and log-compressed. Using the **RGB** color coding, the filter output was coded. The **R**, **G**, and **B** channels were normalized to the range from 0 to 1. The lower frequency backscattered signal (**G***H*_2_) was allocated to the red (**R**) channel, the higher frequency backscattered signal (**G***H*_8_) was allocated to the blue (**B**) channel, and the original unfiltered signal was allocated to the green (**G**) channel. Then, the **RGB** values of each pixel were composed to achieve H-scan ultrasound imaging.

By adjusting the parameter factors of **G***H*_2_(*bt*/*τ*) and **G***H*_8_(*at*/*τ*), where *a* and *b* were parameters for center frequency shifting, the **G***H*_2_ and **G***H*_8_ filters would contain the low and high frequency components of RF signals as much as possible, and the overlap between **G***H*_2_ and **G***H*_8_ would be as small as possible.

In order to better describe the difference between **G***H*_2_ and **G***H*_8_ convolutional filtering results, the values of the **R** and **B** components on each pixel were compared and displayed with the larger one, while the values of the **G** channel were set to zero, so as to emphasize the echo signal from the dominant channel. Thus, an H-scan image displaying the dominant hue was produced. The dynamic range was set to 50 dB.

### 3.3. Statistical Analysis

To determine whether the H-scan ultrasound imaging was useful in breast tumor classification, several test methods were used to analyze the parameters of **RGB** channels. First, the Kolmogorov–Smirnov test was applied to test whether the parameter value distribution of benign and malignant groups was normal. If the distribution was not normal, the Mann–Whitney U test was used; otherwise, the independent-samples T test was used. The performances of each **RGB** channel to discriminate between benign and malignant tumors were evaluated using the receiver operating characteristic (ROC) curve. The area under the ROC curve (Az) was used as the performance index. The *p*-values of all statistical tests less than 0.05 were considered indicators of a significant difference. The ROC analysis and box plots were performed using SigmaPlot (version 13 for Windows; London, UK), and other statistical analysis was conducted using SPSS (version 19 for Windows; SPSS, Chicago, IL, USA).

## 4. Results

**Figure 3:**
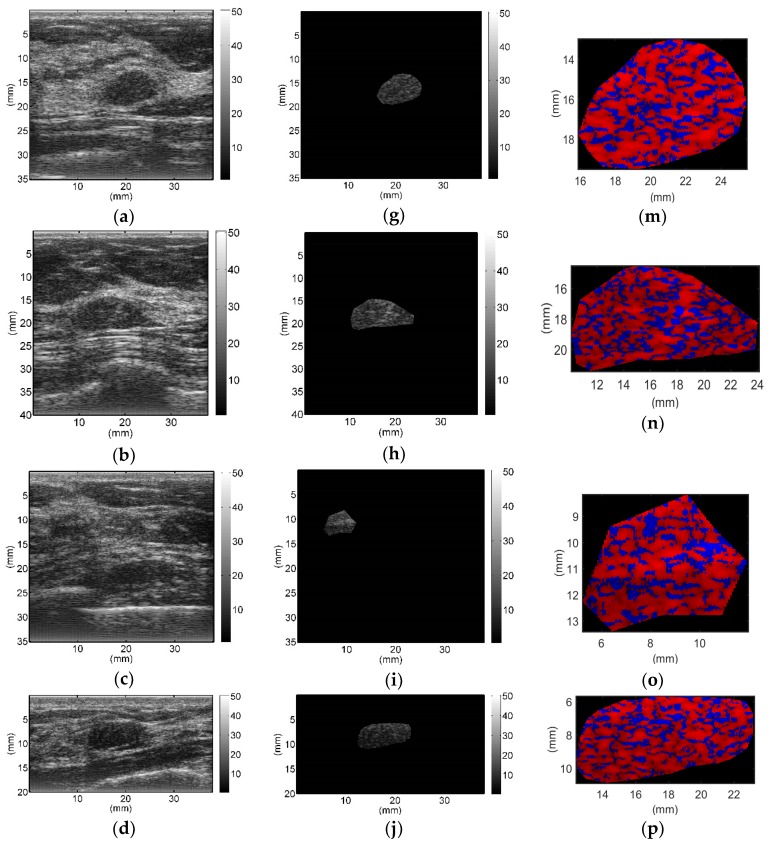

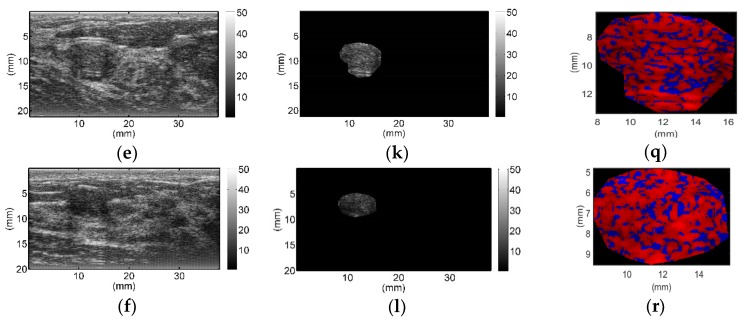
Typical B-mode (**a**–**f**), region of interest (**g**–**l**), and H-scan (**m**–**r**) ultrasound images of benign breast tumors. H-scan images (**m**–**r**) correspond to the regions of interest of B-mode images (**g**–**l**).

**Figure 4:**
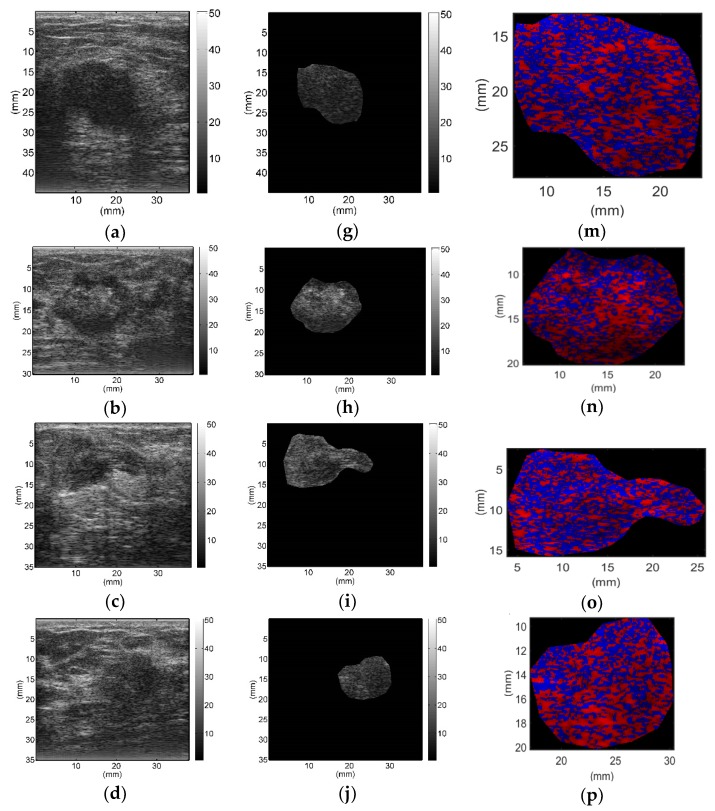

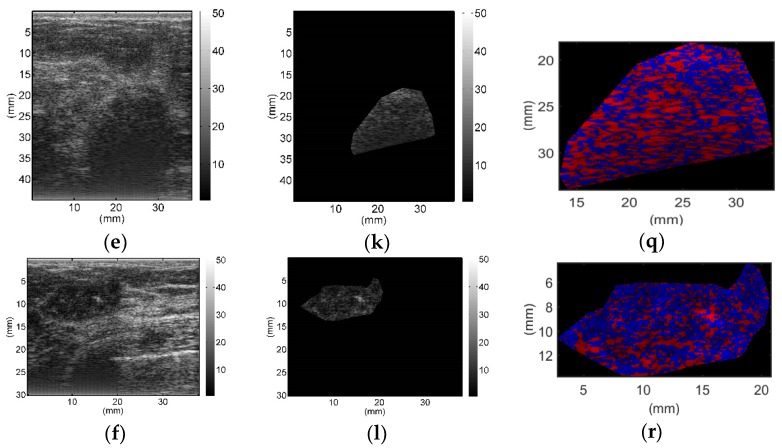
Typical B-mode (**a**–**f**), region of interest (**g**–**l**), and H-scan (**m**–**r**) ultrasound images of malignant breast tumors. H-scan images (**m**–**r**) correspond to the regions of interest of B-mode images (**g**–**l**).

All breast lesions were imaged by H-scan and conventional B-mode ultrasound. Typical B-mode and H-scan images of benign breast tumors are shown in Figure 3, and those of malignant breast tumors are shown in Figure 4. For benign breast tumors, the red (**R**) component is significantly higher than the blue (**B**) component, and most of the red areas adhere to each other, showing a large-scale massive distribution. In contrast, the blue component is more obvious in malignant breast lesions, and the red component mostly shows a small-scale scattered distribution.

**Figure 5:**
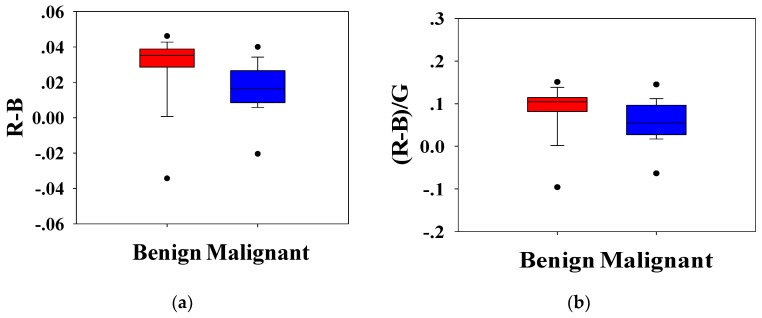

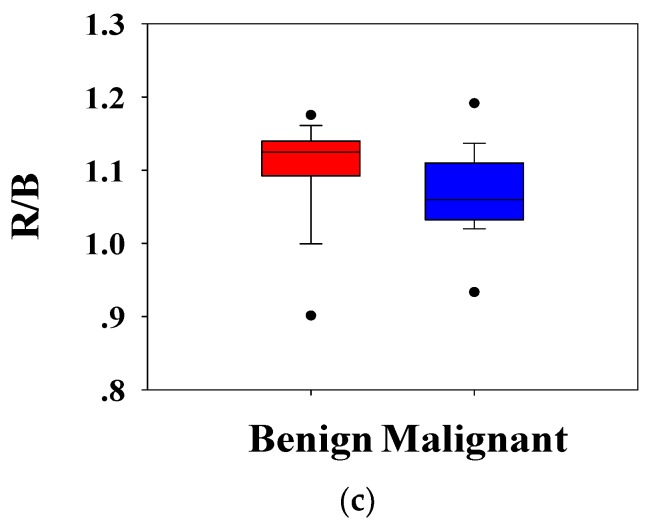
Box plots of $\bar{R-B}$ (**a**), $(\bar{R}-\bar{B})/G$ (**b**), and $\bar{R}/\bar{B}$ (**c**) of H-scan ultrasound images for benign and malignant breast lesions. The *p*-values using the Mann–Whitney U test for (**a**–**c**) were smaller than 0.001, and the group value of benign and malignant breast tumors came from different distributions (*p*-values < 0.01).

Statistical analysis was conducted to analyze the H-scan imaging results. The boxplots for the classification of benign and malignant tumors according to the relationship between **RGB** channels are shown in Figure 5. Figure 5a depicts the magnitude of the red channel minus the magnitude of the blue channel from each pixel, $\bar{R-B}$ (bar indicated average), which is a measure of the relative strength of the two channels. Similarly, Figure 5b shows the ratio of the difference between **RB** channels to the **G** channel, $(\bar{R}-\bar{B})/G$. Figure 5c shows the ratio of the **R** channel to the **B** channel, $\bar{R}/\bar{B}$. It can be seen that the distribution of benign and malignant breast tumors is different. Compared with benign lesions, malignant breast masses had a lower distribution. The Mann–Whitney U test showed that the *p*-values of each distribution were less than 0.001, and the group value of benign and malignant breast tumors came from different distributions (*p*-values < 0.01). It was implied that $\bar{R-B}$, $(\bar{R}-\bar{B})/G$, and $\bar{R}/\bar{B}$ could effectively distinguish benign and malignant tumors.

**Figure 6:**
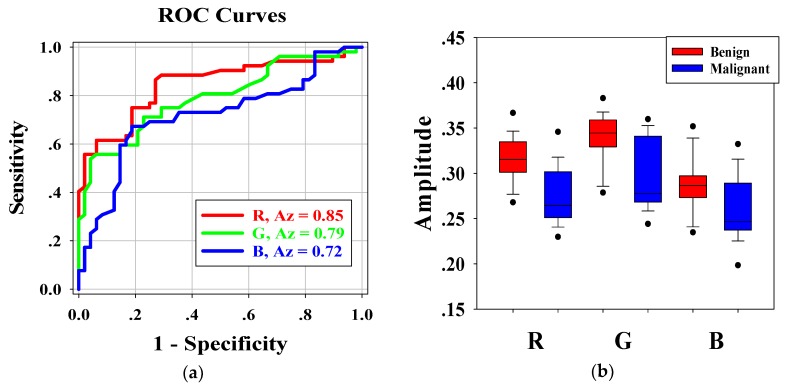
(**a**) The receiver operating characteristic (ROC) curves and the areas under the ROC curves (Az) of the **RGB** channels. (**b**) Boxplots of the average pixel values of the **RGB** channels.

Figure 6a shows the ROC curves for the average pixel values of **RGB** channels of each H-scan image. The Az-values for **R**, **G**, and **B** channel were 0.85, 0.79, and 0.71, respectively. Furthermore, the *p*-values of **RGB** channels were less than 0.001. The results indicated that distribution of **RGB** channels in benign and malignant breast lesions were feasible for classifying benign and malignant breast tumors. According to Az-values, the **R** channel was higher than the other two channels. Hence, the **R** channel was regarded as the best choice for breast tumor diagnosis.

Figure 6b shows the boxplots of the distribution of **RGB** channels in benign and malignant breast tumors. Kruskal–Wallis analysis showed that there were significant differences in the average pixel values of **RGB** channels between benign and malignant breast tumors, and the *p* value of each channel was less than 0.001. Pairwise comparisons of **RB** channels, **GB** channels, and **RG** channels in benign breast tumors showed that the *p*-values were 0.002, <0.001, and 0.001, respectively. Those of malignant breast tumors showed that the *p*-values were 0.046, <0.001, and 0.008, respectively. There were significant differences between **RGB** channels.

## 5. Discussion

The structure of the human body is extremely complex. Different organs and tissues have different acoustic characteristics. Therefore, the acoustic impedance of normal tissue and lesion will be significantly different. Therefore, when the ultrasound signal is transmitted to the human body, different echo information will exist. Compared with conventional B-mode ultrasound, H-scan imaging can show the relative size and spatial distribution of different scatterers and more abundant information of breast lesions can be obtained by processing the RF signals and estimating the scatterers in the ROI according to the frequency dependence of different sizes of scatterers.

To the best of our knowledge, this study is the first to employ H-scan ultrasound imaging to distinguish benign and malignant breast tumors. The characteristics of H-scan ultrasonography in benign and malignant breast lesions were compared, as shown in Figure 3 and Figure 4. For benign breast tumors, more red components were shown, while for malignant breast tumors, blue components were more significant.

The red component is related to the low frequency information and represents the scatterer with a larger diameter, while the blue component is related to the high frequency information and represents the scatterer with smaller diameter [19,20,21,22,23]. The brightness of the red and blue components reflects the echo intensity of different scatterers. It can be concluded that malignant breast lesions are more related to the smaller diameter of the microstructure. The statistical analysis of **RGB** channels further demonstrated the difference of scatterer size distribution between benign and malignant breast tumors, which could be used as an important indicator to differentiate between benign and malignant breast tumors. Therefore, it is feasible to distinguish benign from malignant breast tumors by analyzing the size of scatterers in breast lesions with H-scan imaging. In addition, by displaying the scatterer distribution and scatterer intensity information of different sizes in color images, H-scan ultrasound imaging may enable clinicians to discover more hidden information that is not observed in conventional B-mode ultrasound, thus providing new reference information for breast tumor ultrasound diagnosis.

It should be noted that some limitations may have impact on the results of this study. With the increase of ultrasound scanning depth, the attenuation of high frequency component is greater than that that of low frequency component. Therefore, compared with **G***H*_2_ matched filter, **G***H*_8_ matched filter loses more useful information due to attenuation. That is, with the increase of depth, the output intensity of the **B** channel becomes weaker than that of the **R** channel. In addition, when adjusting **G***H*_2_ and **G***H*_8_ matched filters, the low frequency and high frequency information of the original signal should be different from each other as much as possible, and the overlapping parts of the matched filters should be minimized to better identify scatterers of different sizes. The adjustment of matched filters is limited by system bandwidth. If the bandwidth is large, the spectrum of the received signal is rich and the loss of high frequency components is small. However, if the bandwidth of the system is too large, the signal-to-noise ratio and sensitivity will be reduced [29].

## 6. Conclusions

This paper proposed a new method based on H-scan ultrasound imaging to classify benign and malignant breast tumors. H-scan ultrasound images showed that benign breast tumors have more red components, while malignant breast tumors have more blue components. Statistical analysis showed that there were significant differences between **RGB** channels of benign and malignant breast lesions. It is concluded that H-scan ultrasound imaging can be used as a new method for classifying benign and malignant breast tumors.
